# Supplementary material for: Statistical and functional convergence of common and rare genetic influences on autism at chromosome 16p
Source: Nat Genet. 2022 Oct 24;54(11):1630–9. doi: 10.1038/s41588-022-01203-y (PMC9649437; doi:10.1038/s41588-022-01203-y)
Supplement: Supplementary file 1 — Supplementary Figs. 1–23, Note and Tables 1–4. [file 41588_2022_1203_MOESM1_ESM.pdf]

nature genetics

Article https://doi.org/10.1038/s41588-022-01203-y

Statistical and functional convergence of common and rare genetic influences on autism at chromosome 16p

In the format provided by the authors and unedited

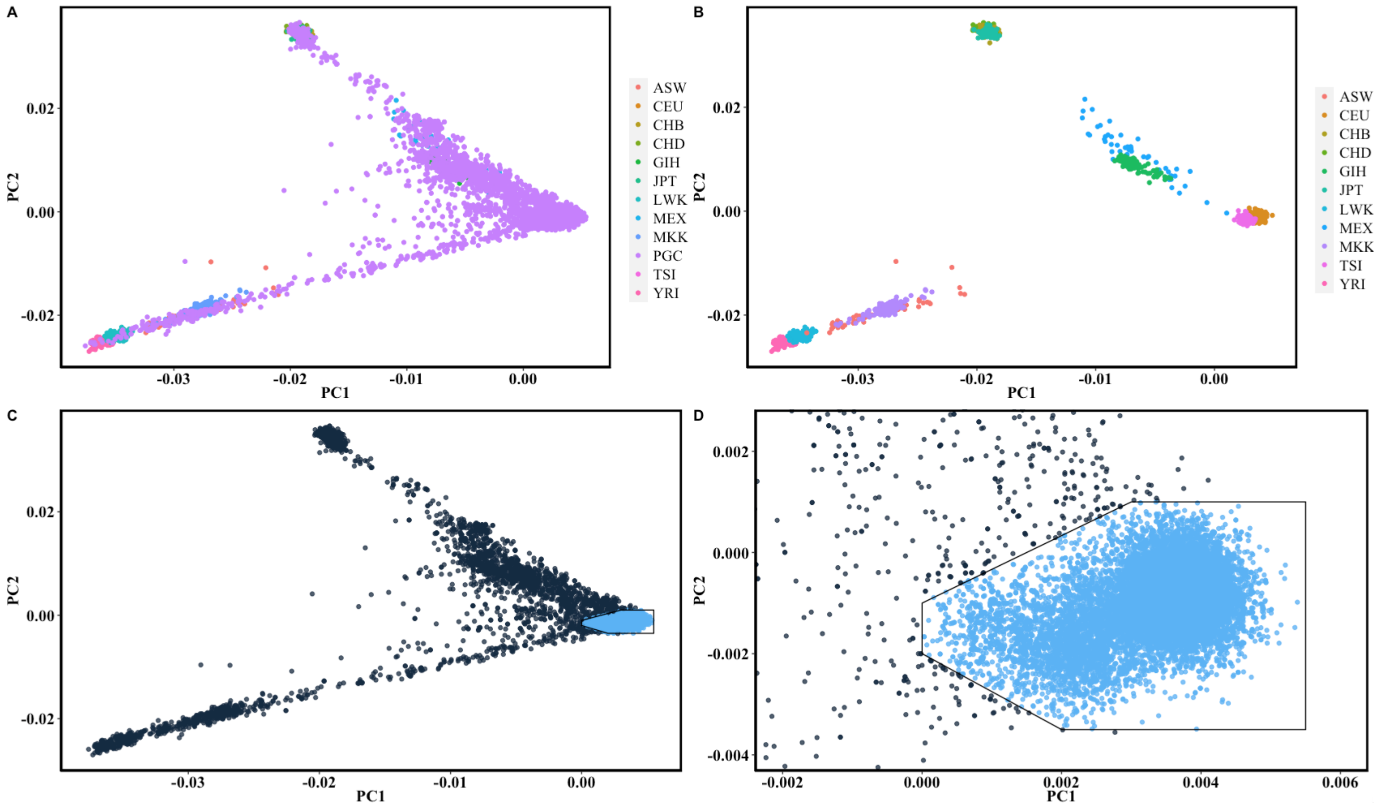

Supplementary Figure 1: Ancestry analysis of PGC ASD trios

(A-B) The ASD trios from the Psychiatric Genomics Consortium Autism group (PGC) are as described previously (Methods) with the exception of the inclusion of all probands from multiplex families. We defined a European ancestry subset of PGC for analysis by generating principal components of ancestry using PLINK and by visual inspection relative to Hapmap reference populations. The figure legend lists Hapmap subpopulations and PGC samples. **(C-D)** We defined a family as European ancestry if both parents and proband were European ancestry by principal component analysis (4,335 of 5,283 trios, 82%).

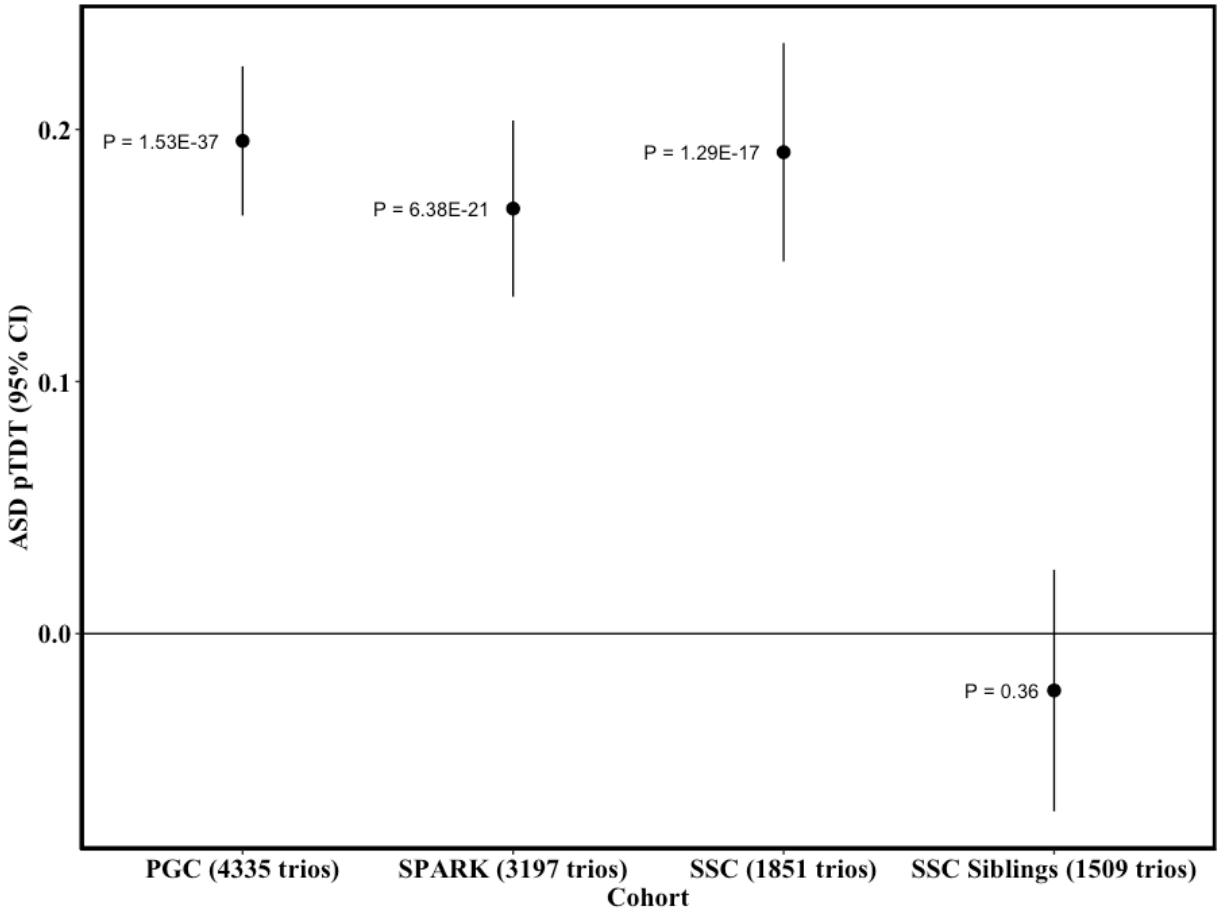

Supplementary Figure 2: pTDT of ASD PGS in three European-ancestry ASD trio cohorts

We performed whole genome ASD pTDT using three independent ASD trio datasets as previously described (see Methods; n = 4,335 case-pseudocontrol pairs from the Psychiatric Genomics Consortium; n = 3,197 ASD trios from SPARK; n = 1,851 trios from SSC, n = 1,509 unaffected-parent trios from the SSC). Point estimates are transmission of ASD PGS in units of standard deviations of the mid-parent PGS distribution. PGS constructed from iPSYCH ASD GWAS (Methods). Error bars are 95% confidence intervals for transmission. P-values are from two-sided one-sample t-tests of the null that transmission equals 0.

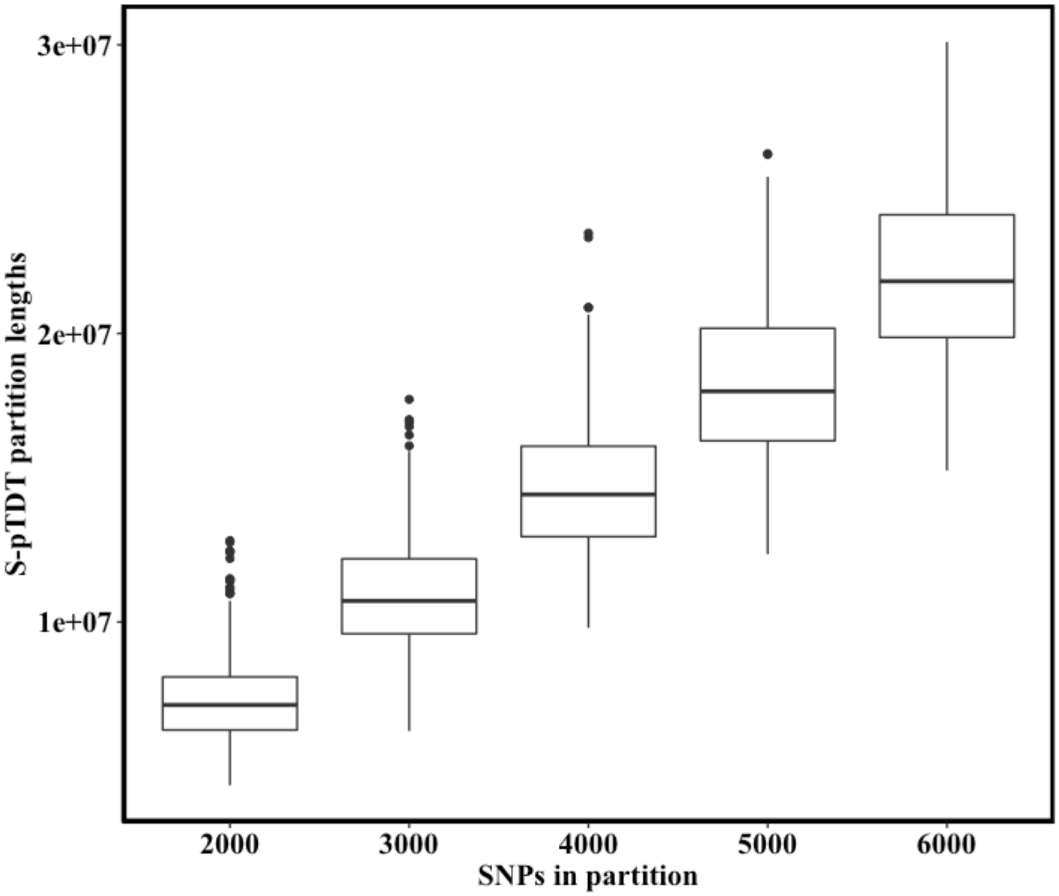

Supplementary Figure 3: S-pTDT partition lengths in base pairs as a function of number of SNPs in partition

The distribution of partition lengths in base pairs as a function of number of SNPs in the partition. If the distance between consecutive SNPs is greater than 1Mb, the counted distance is reduced to 1Mb (Methods). We plot here both forward and reverse partitions (i.e. starting at the beginning and end of chromosomes) of count 2,000 SNPs (n = 704), 3,000 SNPs (n = 464), 4,000 SNPs (n = 346), 5,000 SNPs (n = 272), and 6,000 SNPs (n = 220). The lower whisker, lower hinge, center, upper hinge and upper whisker correspond to lower hinge minus 1.5 multiplied by the interquartile range, the 25th percentile, the median, the 75th percentile, and the upper hinge plus 1.5 multiplied by the interquartile range, respectively.

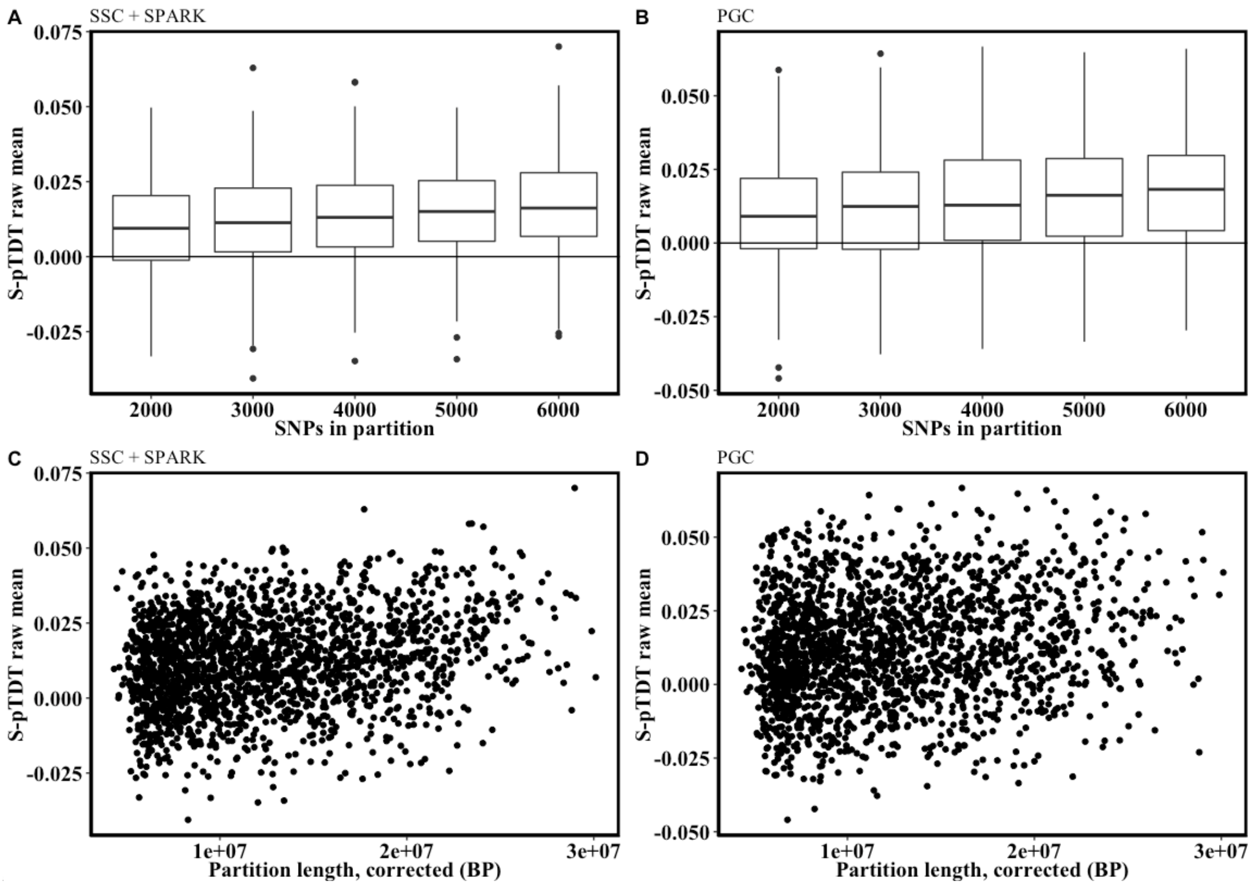

Supplementary Figure 4: Mean ASD S-pTDT as a function of number of SNPs in partition and partition base pair size in SSC+SPARK and PGC ASD trios

The relationship between number of SNPs in partition, corrected partition length, and S-pTDT transmission. **(A-B)** S-pTDT increases with the number of SNPs in partition in analysis of the SSC + SPARK cohorts (n = 5,048 trios) and the PGC (n = 4,335 trios). The lower whisker, lower hinge, center, upper hinge and upper whisker correspond to lower hinge minus 1.5 multiplied by the interquartile range, the 25th percentile, the median, the 75th percentile, and the upper hinge plus 1.5 multiplied by the interquartile range, respectively. **(C-D)** S-pTDT increases with corrected partition length in the SSC + SPARK cohort and the PGC cohort. S-pTDT in units of standard deviations of the mid-parent PGS distribution.

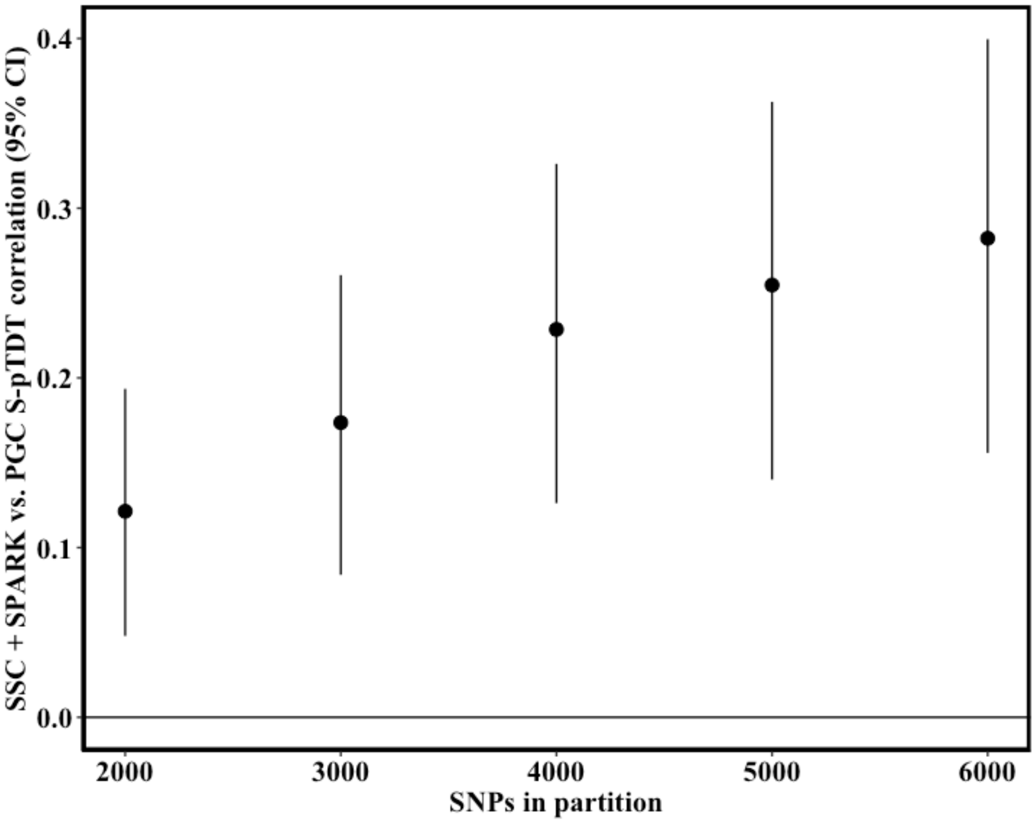

Supplementary Figure 5: S-pTDT correlation between SSC+SPARK and PGC trios across partition sizes.

The correlation between S-pTDT values in SSC+SPARK (n = 5,048 trios) and in PGC (n = 4,335 trios). The correlation analysis includes both forward and reverse partitions. The cross-cohort correlation increases with the number of SNPs in partition. Error bars are 95% confidence intervals on the mean of the S-pTDT transmission.

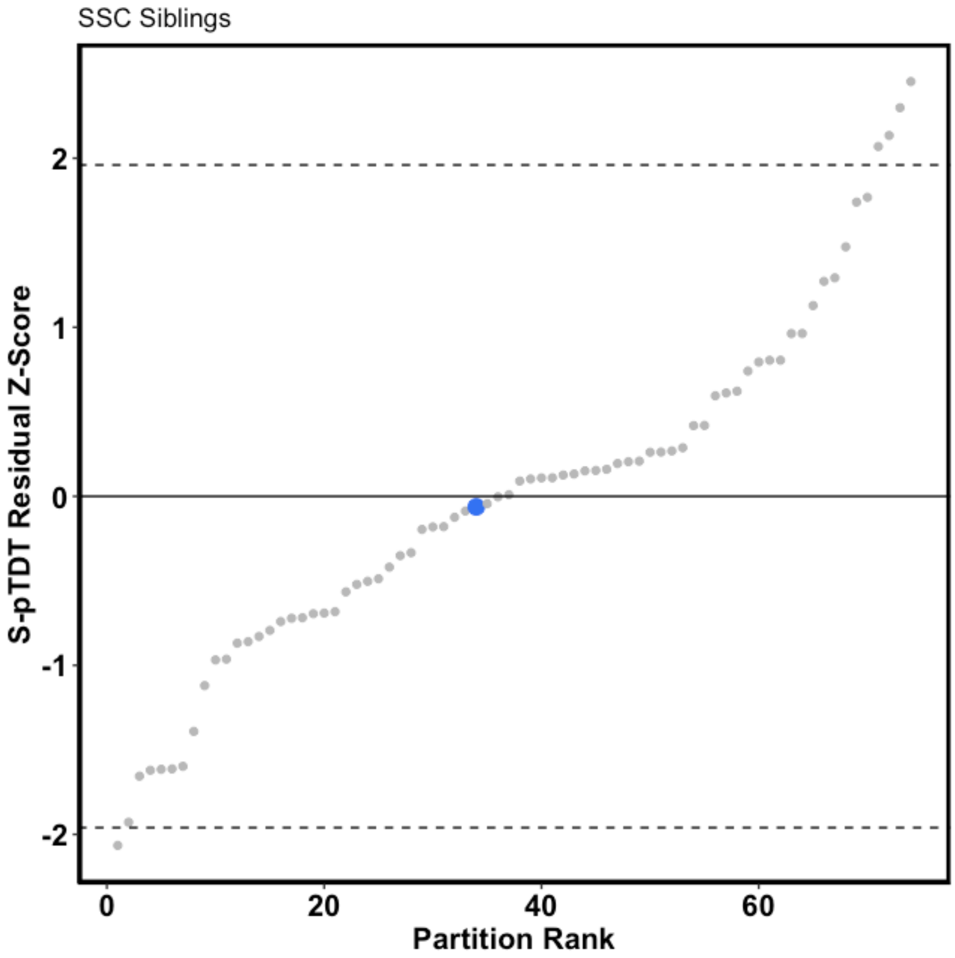

Supplementary Figure 6: 16p ASD PGS is not over-transmitted to 1,509 unaffected siblings in SSC.

An identical analysis to that presented in Figure 1D, except instead of testing for transmission of 16p PGS from parents to probands, here we test transmission from parents to unaffected siblings. The blue dot denotes the 16p partition, while the other gray dots are the 73 other control partitions. The y-axis is the S-pTDT z-score.

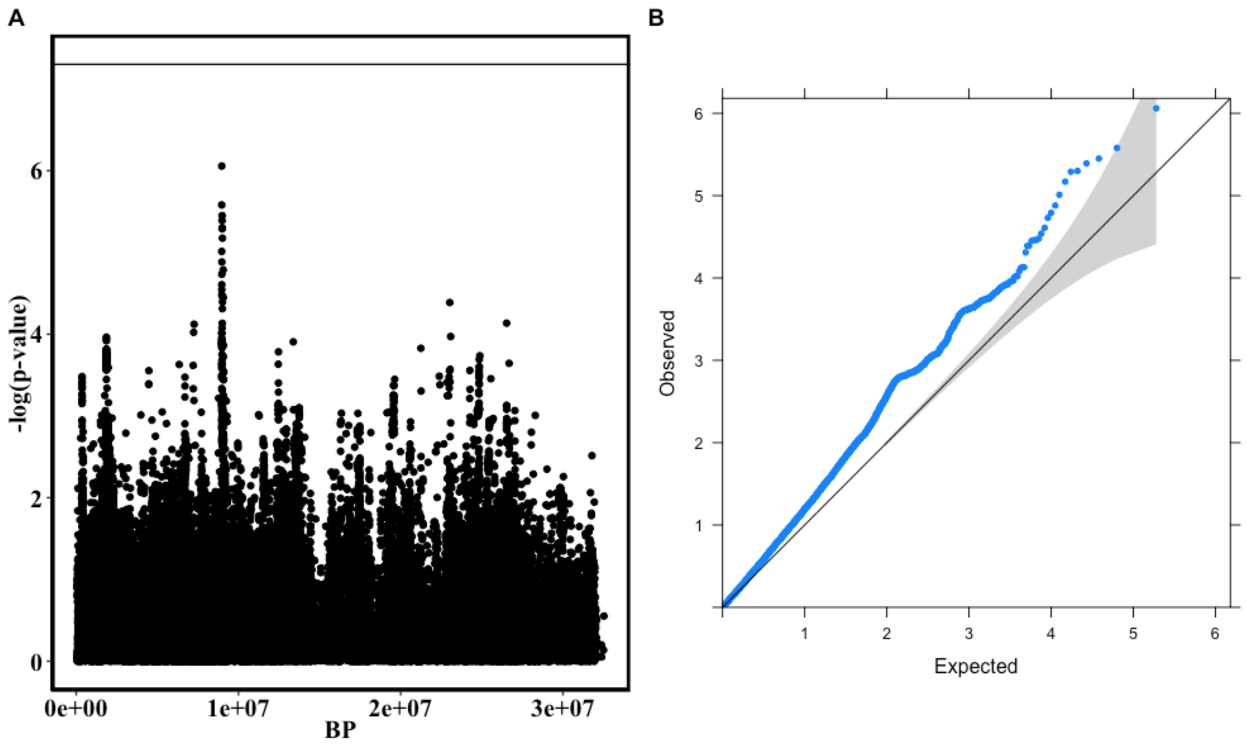

Supplementary Figure 7: ASD GWAS at 16p

(A) Local manhattan plot visualization of ASD GWAS on 16p. Each point is a SNP, with the x-axis value its position on chromosome 16 and the y-axis value the -$\log_{10}$(P-value) from the GWAS. The GWAS is an meta-analysis of the iPSYCH and PGC collections (26,067 cases, 46,455 controls). **(B)** The same SNPs as (A), here represented as a quantile-quantile plot (code adapted from Matthew Flickinger, https://genome.sph.umich.edu/wiki/Code_Sample:_Generating_QQ_Plots_in_R). Confidence interval represents alpha = 0.05.

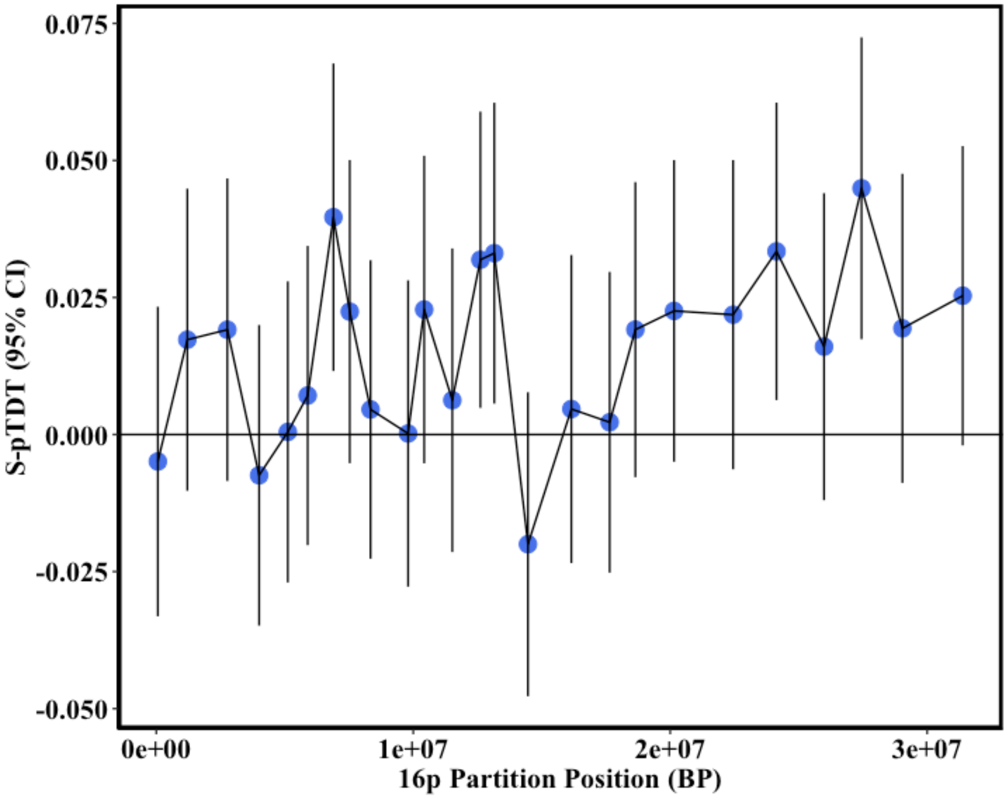

Supplementary Figure 8: ASD S-pTDT in SSC + SPARK in LD-independent blocks at 16p

We performed S-pTDT in 25 LD-independent blocks on chromosome 16p using the SSC + SPARK trios (n = 5,048 trios) and the iPSYCH-only ASD GWAS. The LD-independent blocks are from a previous publication (Methods). The blue dots are the mean S-pTDT value in SSC + SPARK using an ASD PGS constructed from SNPs in that block, in units of SDs of the mid-parent PGS distribution. The x-axis position of the blue dots is the midpoint of the block. The error bars are 95% CI of the S-pTDT mean.

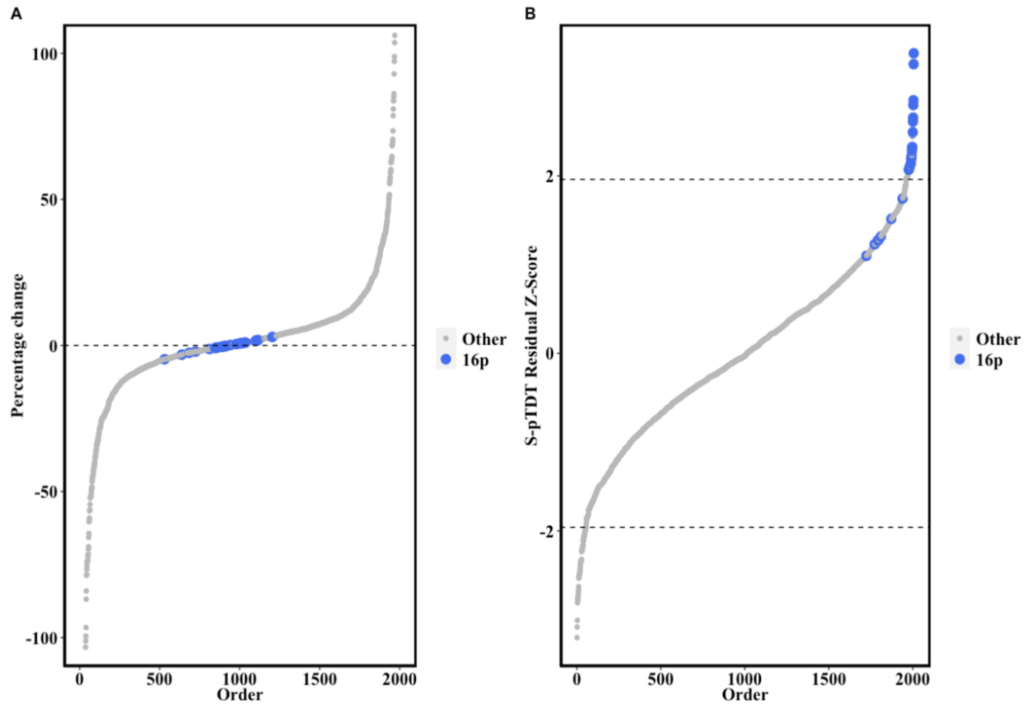

Supplementary Figure 9: ASD S-pTDT without SSC+SPARK probands carrying 16p CNV

We tested the hypothesis that ASD probands with a neurodevelopmental-disorder associated CNV on 16p in SSC + SPARK drive the S-pTDT signal by identifying these probands and removing them from analysis. We defined genomic-disorder associated CNV based existing literature curation (Methods). Of the 5,048 trios in the SSC + SPARK analysis, we removed 51 (1.0%) where a proband carried at least one of this class of CNV (either inherited or *de novo*) on the p-arm of chromosome 16. **(A)** S-pTDT estimates do not qualitatively change after removal of these 51 trios, with small percentage change in S-pTDT estimates for the 16p partitions (blue) relative to the change in other partitions (gray). **(B)** We also find that the 16p partitions remain with large residual z-scores after removal of these trios.

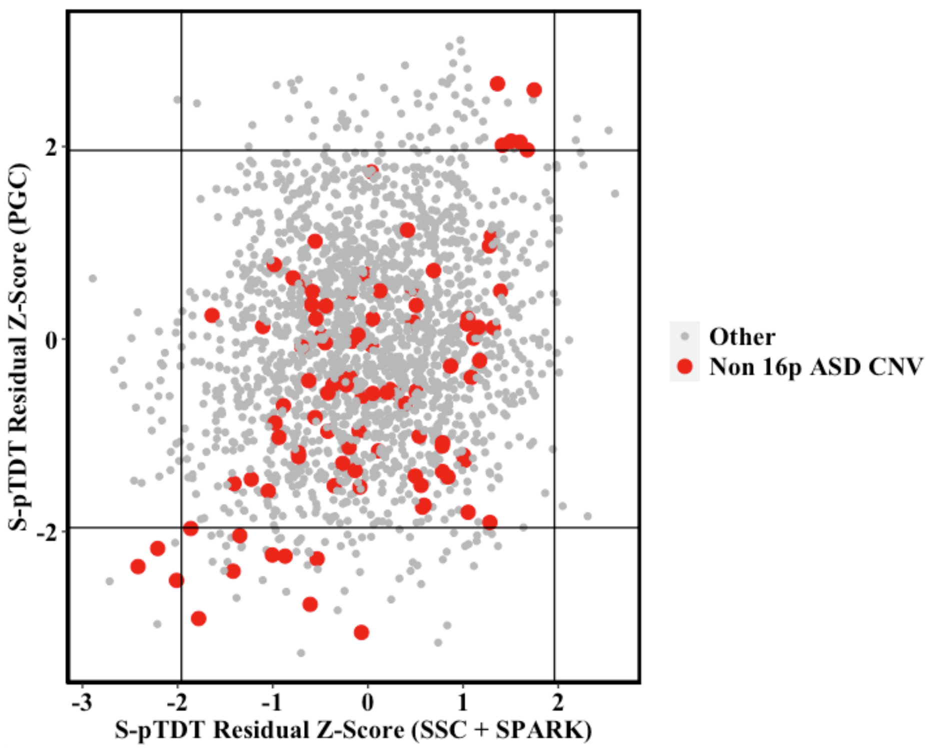

Supplementary Figure 10: ASD S-pTDT highlighting partitions including ASD-associated CNV

Primary ASD S-pTDT result (Figure 1B), highlighting partitions in red that include an ASD-associated CNV. We defined ASD-associated CNV as those listed in SFARI Gene (Methods). The axes are residual z-scores from the S-pTDT model (Methods), for SSC+SPARK (x-axis) and PGC (y-axis).

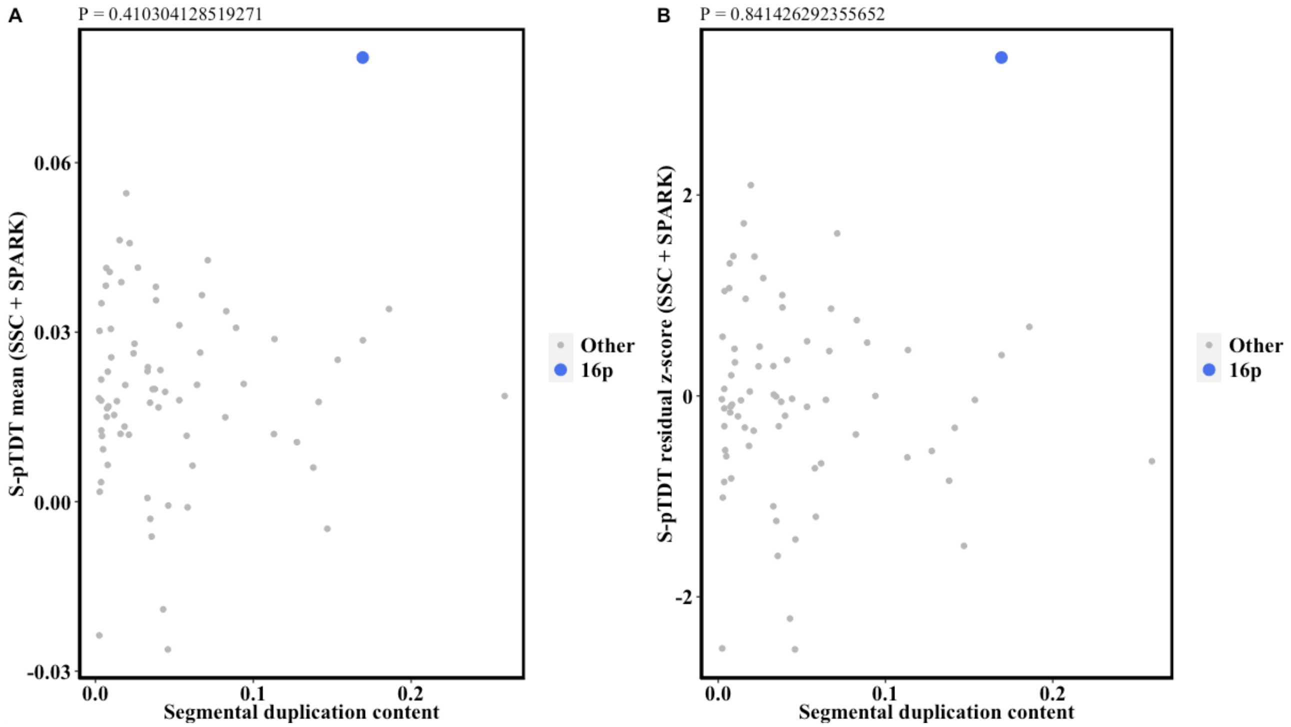

Supplementary Figure 11: Association between segmental duplication content and ASD S-pTDT

(A) Association between segmental duplication content per 33Mb partition and ASD S-pTDT mean in SSC+SPARK. The p-value at the top of the plot is from the Pearson correlation of segmental duplication content and S-pTDT mean. The 16p partition is noted in blue. **(B)** As in (A), except the y-axis is now the residual z-score from the ASD S-pTDT model (Methods).

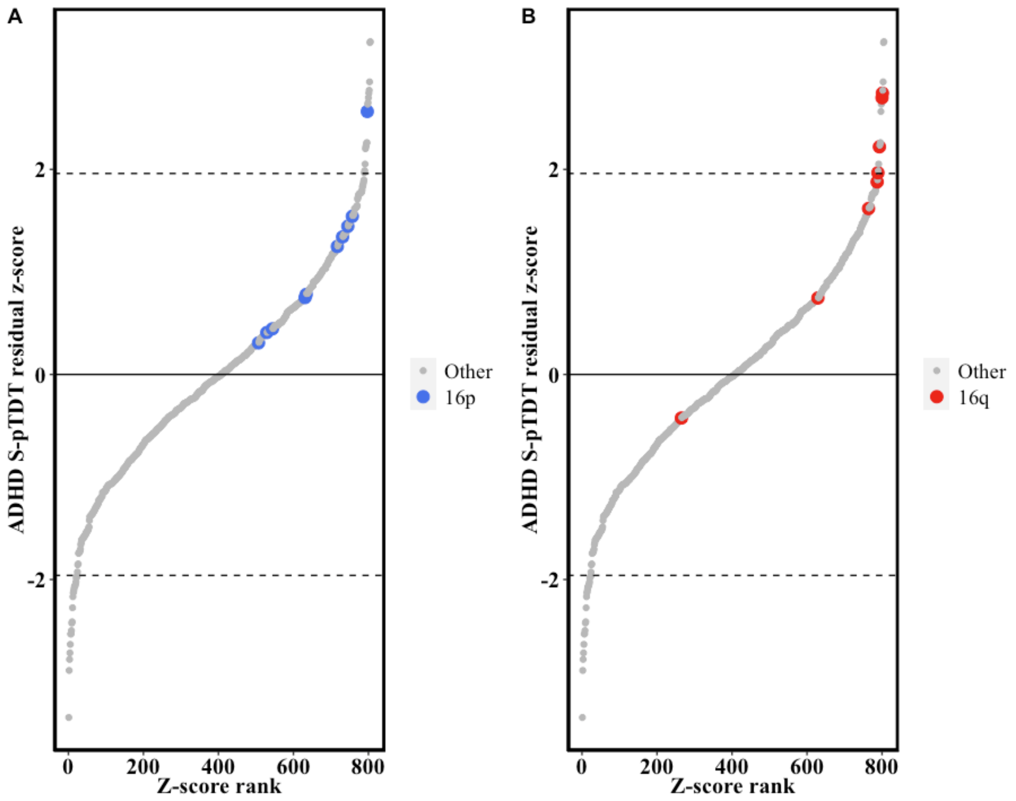

Supplementary Figure 12: ADHD S-pTDT

(A) We performed S-pTDT using ADHD trios (n = 1,634 European ancestry trios from the PGC) and an ADHD PGS generated from a non-overlapping ADHD GWAS from the iPSYCH collection (25,895 cases, 37,148 controls). The partitions located on 16p are denoted in blue. The 16p partition with marginal significance (5,000 SNPs, start BP: 28,861,734, end BP: 69,367,996, residual z-score: 2.56) has 4,807/5,000 (96%) of SNPs beyond the 16p boundary, suggesting that it reflects signal on 16q. **(B)** This hypothesis is confirmed when we highlight the position of 16q partitions, many of which are marginally significant in 16q.

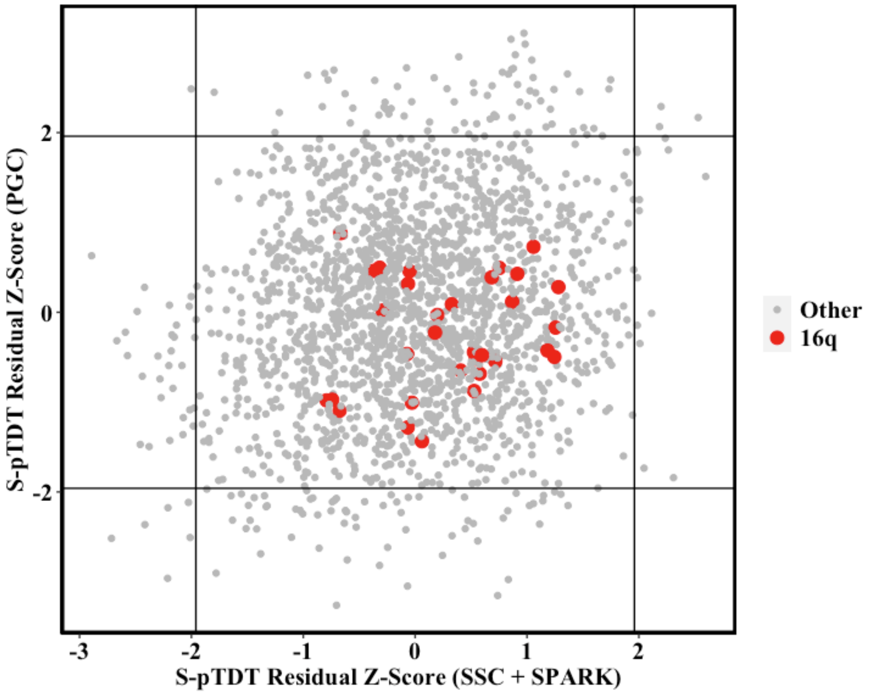

Supplementary Figure 13: ASD S-pTDT for partition on 16q

Primary ASD S-pTDT association result (Figure 1B), with partitions in red on chromosome 16 that are not located on the p-arm (start position > 33Mb). As opposed to partitions on the p-arm, partitions on the q-arm are not enriched in high S-pTDT values. The axes are residual z-scores from the S-pTDT model (Methods), for SSC+SPARK (x-axis) and PGC (y-axis).

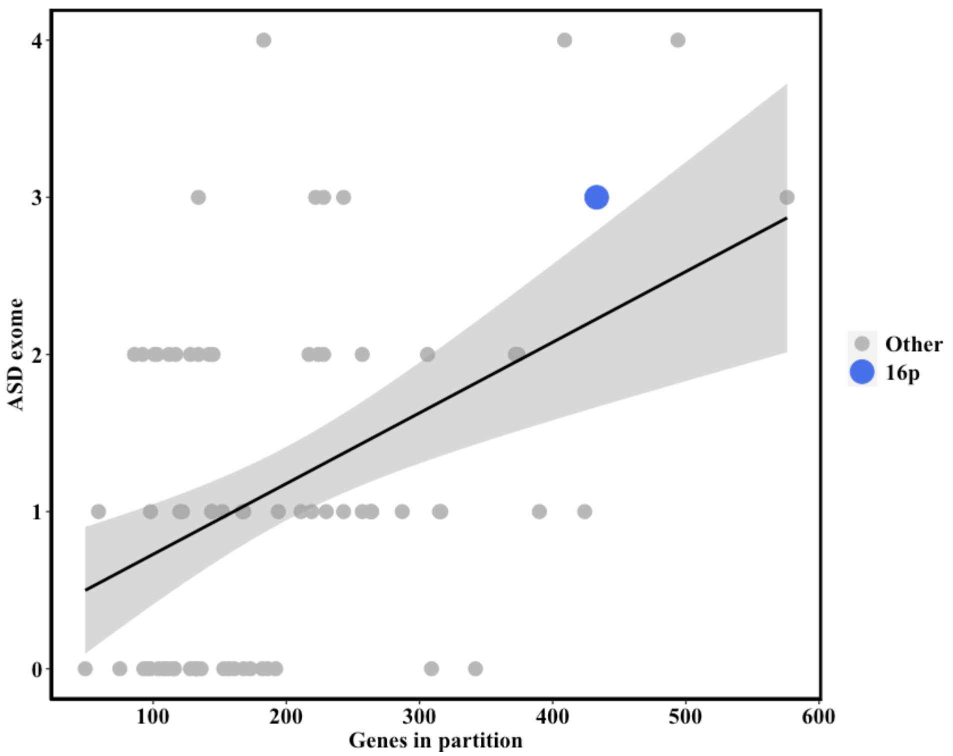

Supplementary Figure 14: Association between total genes and genes implicated in ASD from exome association studies

Across 33Mb partitions, the relationship between the number of genes in partition (x-axis) and the number of genes implicated in ASD via rare variant exome-association studies (TADA q-value < 0.1) (y-axis).[1] Each point is a 33Mb region, with the 16p region highlighted in blue. The shaded region denotes a 95% CI. The trend line is a linear best fit and the shaded regions denote a 95% CI.

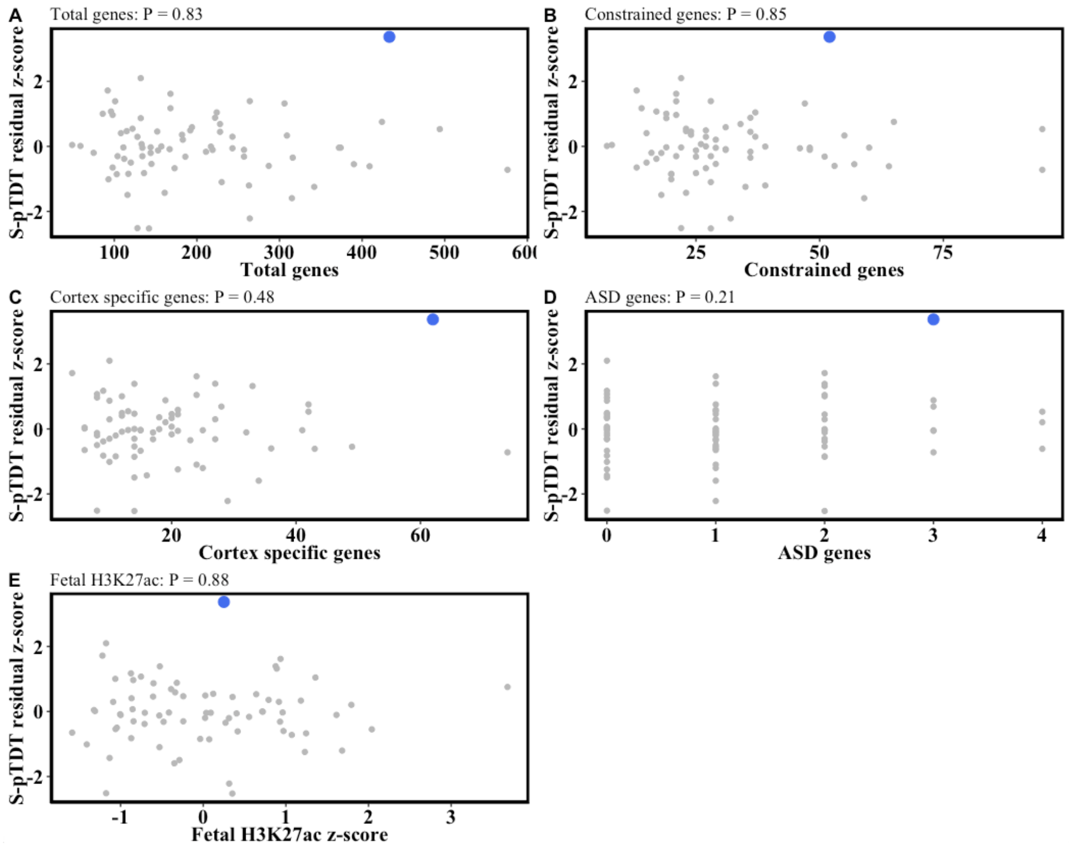

Supplementary Figure 15: Association between gene/enhancer density and S-pTDT

No association between ASD S-pTDT signal (residual z-score) and markers of region gene density for the 33Mb partitions. P-values above plots are from the x-y Pearson correlations (two-sided). **(A)** There is no relationship between the number of genes in the partition and normalized S-pTDT signal (P = 0.83). **(B)** There is no relationship between the number of constrained genes (probability of loss-of-function intolerance ≧ 0.9) and normalized S-pTDT signal (P = 0.85). **(C)** There is no relationship between the number of genes specifically expressed in cortex (defined as top 10% highest cortex vs. non-brain tissue t-statistic[2]) and S-pTDT signal (P = 0.48). **(D)** There is no relationship between the number of genes in the partition implicated in ASD via rare variant exome-association studies (Methods) and the S-pTDT signal (P = 0.21). **(E)** There is no relationship between the density of accessible fetal brain enhancers S-pTDT signal (P = 0.88) (Methods).

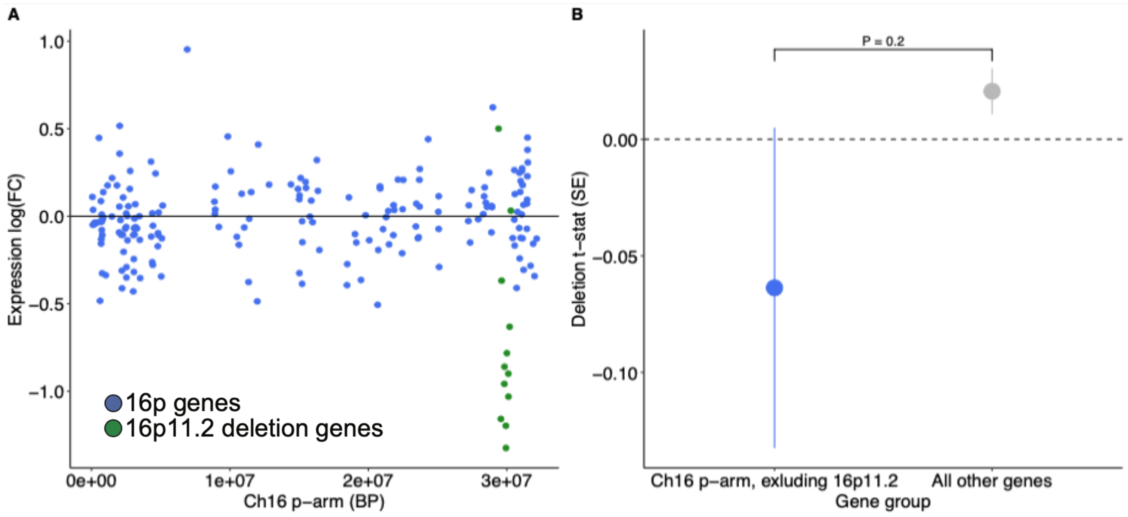

Supplementary Figure 16: Effect of *in vitro* deletion of 16p11.2 on genes with low neuronal expression. (A) Differential expression of n = 189 genes on 16p with neuronal expression below median for all genes. Differential expression log_2 fold-change for genes on 16p not different from 0 (mean = -0.004, P = 0.78, differential expression t-statistic -0.06, P = 0.35). Mean of the differential expression t-statistic distribution for these genes not different from all other genes (P = 0.2). Genes in the deletion region +/- 0.1Mb are green, while all other genes on 16p are in blue. The y-axis is the log_2 fold-change per gene. **(B)** Mean of the differential expression t-statistic distribution for these genes not different from all other genes (P = 0.2, two-sided two-sample t-test)

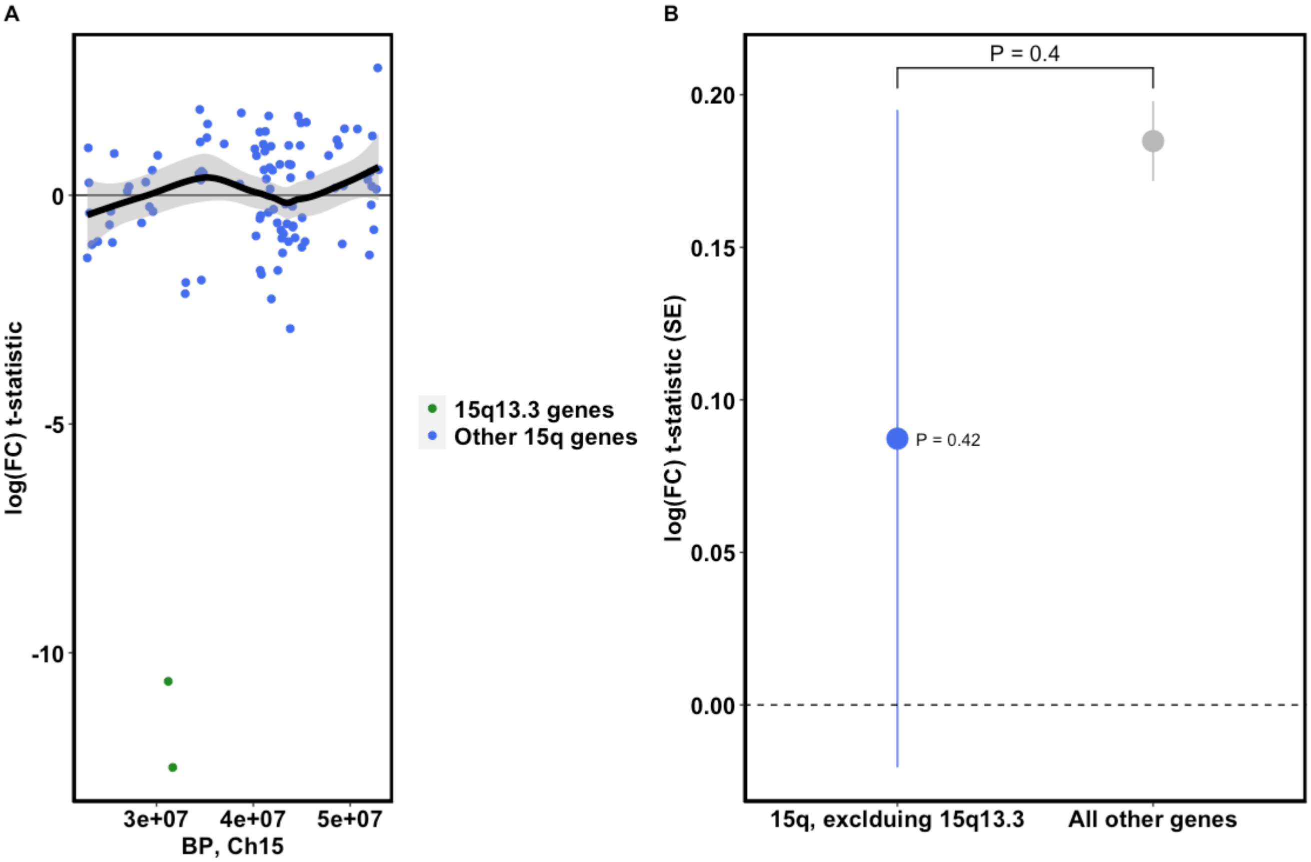

Supplementary Figure 17: *In vitro* deletion analysis of 15q13.3. Induced pluripotent stem cells undergo CRISPR-Cas9-mediated deletion of the 15q13.3 CNV region, differentiation into induced neurons and transcriptome profiling with RNA-seq (n = 11 biological replicates). Differential expression analysis compares these samples to controls (n = 6 biological replicates) without deletion of the locus. **(A)** Differential expression of 15q genes after deletion of 15q13.3 locus, with analysis restricted to genes with above median normalized expression level over all samples in analysis. Genes in the deletion region +/- 0.1Mb are green, while all other genes on 15q are in blue. The y-axis is the log_2(fold-change) t-statistic per gene. The trend line is for genes outside of the CNV (blue dots) with a 95% CI shaded in gray. **(B)** 15q13.3 deletion effect on 15q genes is not different from its effect on all other genes. Point estimates are of mean differential expression t-statistic for the group of genes +/- SE. Comparison P-value is from two-sided two-sample t-test comparing group distributions.

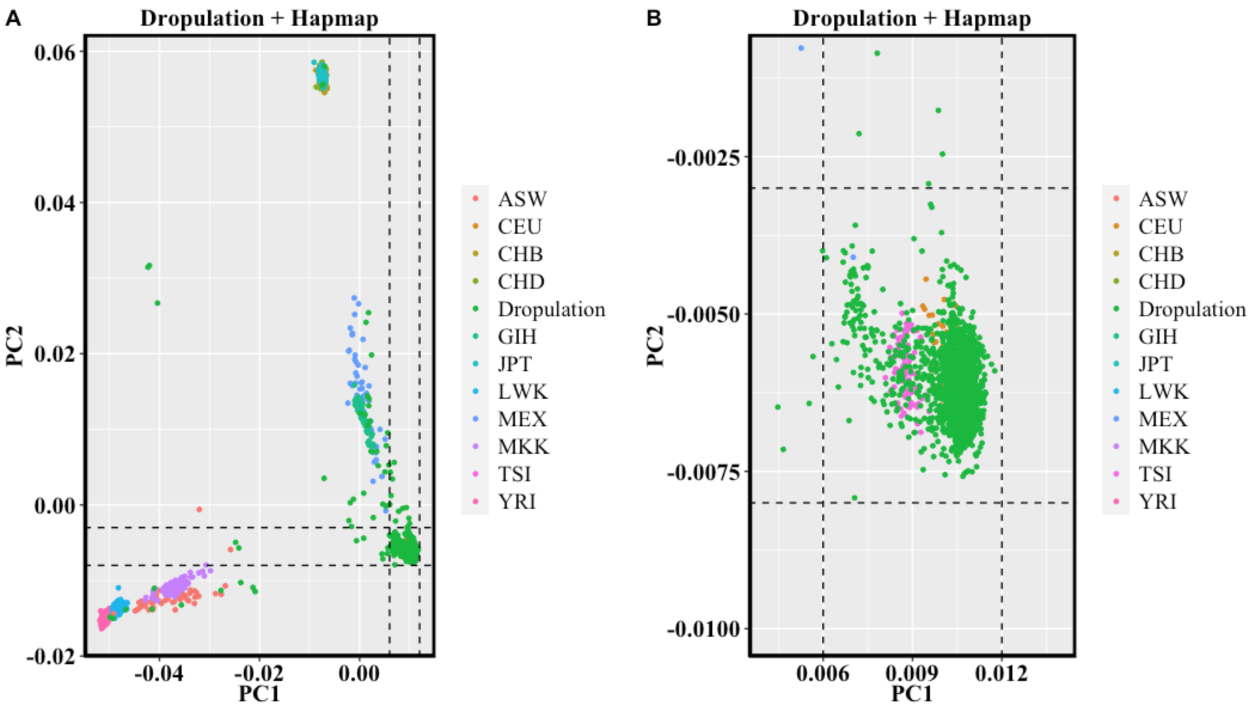

Supplementary Figure 18: Ancestry analysis of single-nucleus RNA-seq donor samples

We performed principal components analysis to identify ancestrally homogeneous subgroups of the single-nucleus RNA-seq donor samples. Since the majority of the samples were of European ancestry by visual inspection of the PCs, we defined a European ancestry subgroup for analysis based on alignment with European ancestry Hapmap samples. We removed regions of long-range LD before computing principal components with PLINK. **(A)** All Hapmap and Dropulation samples in principal component space, colored by group. Abbreviations in the legend correspond to Hapmap subgroups. Dropulation samples in the dashed square are defined as European ancestry and retained for downstream analysis (1707/1770 samples, 96%) **(B)** Same as (A), magnified.

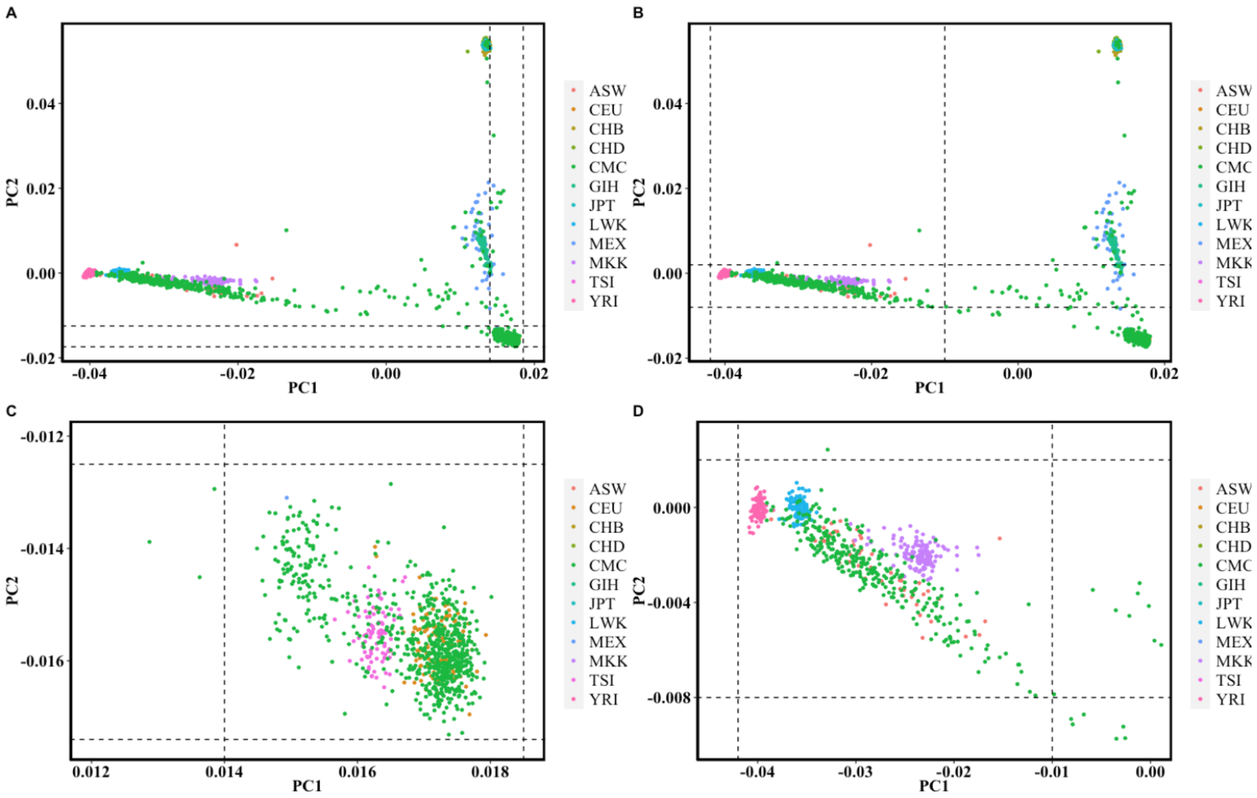

Supplementary Figure 19: Ancestry analysis of CommonMind samples

We performed principal components analysis to identify ancestrally homogeneous subgroups of the CommonMind samples. We merged the CommonMind samples with Hapmap samples for ancestral reference and removed regions of long-range LD before calculating principal components of ancestry using PLINK. **(A,C)** We defined a European ancestry subgroup of the CommonMind samples by visual inspection of alignment with Hapmap samples; we retained samples within the dashed boundaries (694/1,076 of the genotyped samples, 64%). **(B,D)** We defined an African ancestry subgroup of the CommonMind samples by visual inspection of alignment with Hapmap samples; we retained samples within the dashed boundaries (299/1,076 of the genotyped samples, 28%).

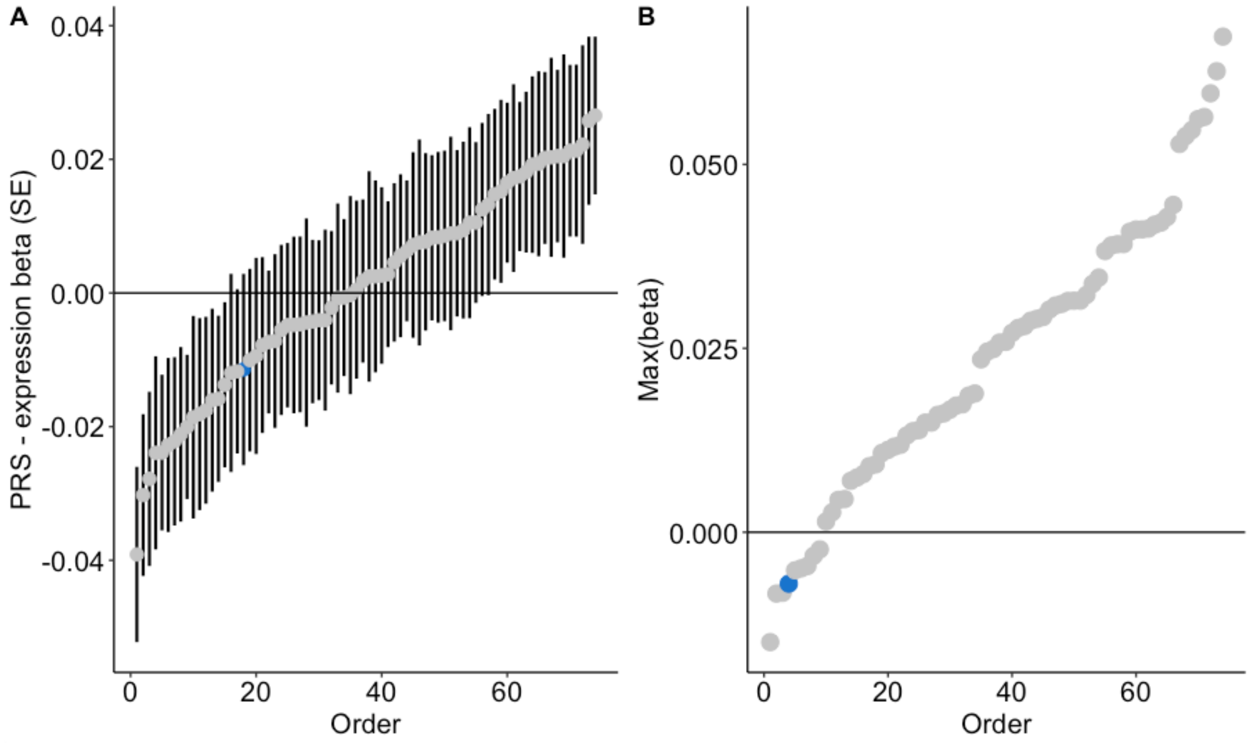

Supplementary Figure 20: Regional ASD PGS - expression associations for genes with lower baseline expression.

Analyses are identical to those in Figure 3B-C, except performed with genes in the bottom half of expression in glutamatergic neurons. (A) Association of regional PGS to mean expression across 74 partitions (n = 544 samples). Error bars are standard error from regression described in Figure 3 and Methods. (B) Most positive association across the three cohorts between mean expression and 16p ASD PGS.

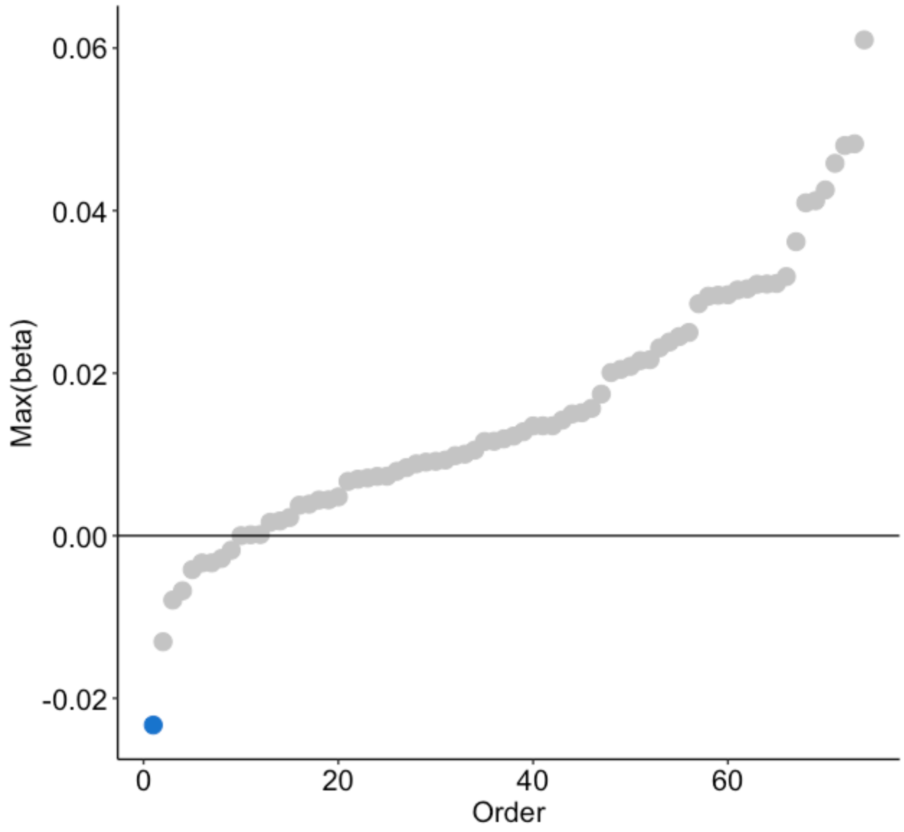

Supplementary Figure 21: Association between regional PGS and mean expression controlling for global principal components of ancestry in each of the three cohorts.

We repeated the analysis in Figure 3C with a distinct control for genetic ancestry. We added 10 genetic principal components to the regression models for each cohort. Each cohort is ancetrally homogeneous and principal components were calculated within each cohort, using PLINK after excluding regions of long-range linkage disequilibrium[3,4]. 16p (blue point) remains the region with the most consistently negative association to its own regional PGS in this sensitivity analysis.

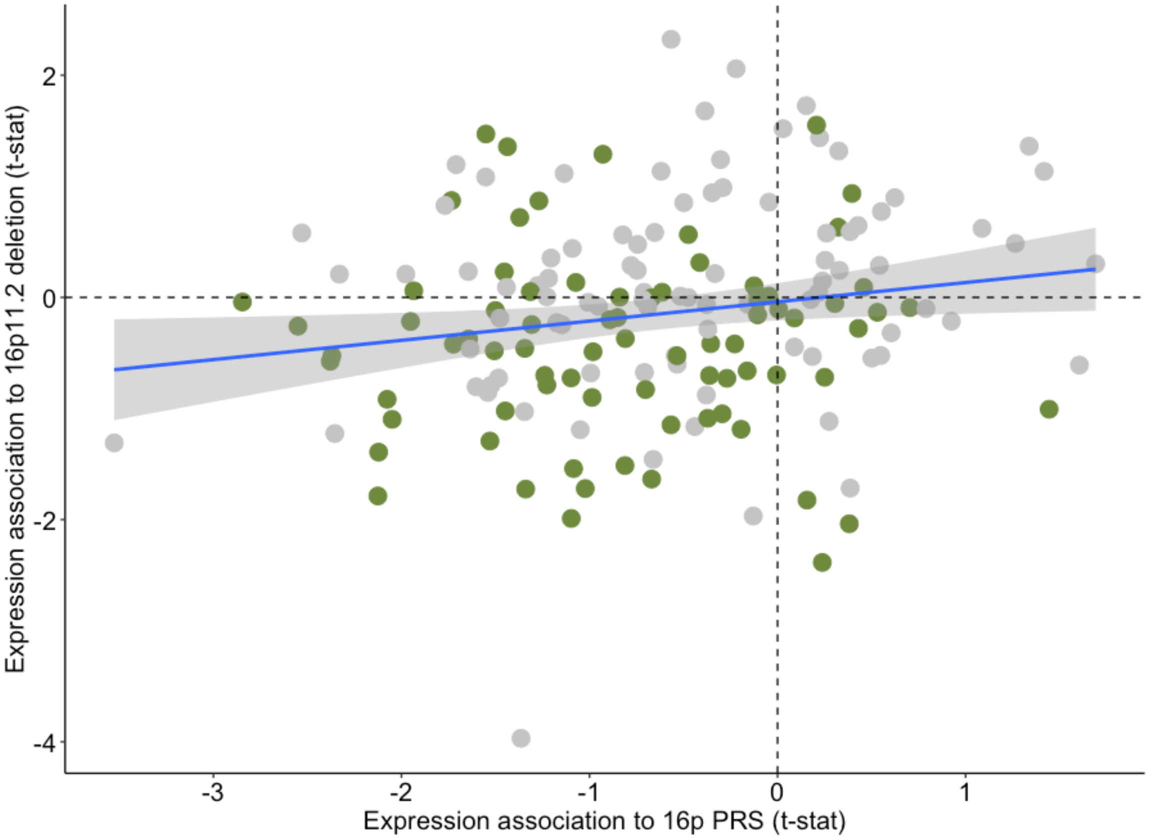

Supplementary Figure 22: Correlation between gene association to 16p PGS and to 16p11.2 *in vitro* deletion

The positive correlation of 16p genes in their association to 16p PGS and to the 16p11.2 *in vitro* deletion. The x-axis shows the association t-statistics from the all sample meta-analysis of 16p PGS and 16p gene expression. The y-axis shows the association t-statistics from the 16p11.2 *in vitro* deletion analysis. The shaded region is 95% CI. This plot is the same as Figure 4A with the inclusion of the outlying point. The green points are in the telomeric region on 16p. The trend line is a linear best fit and the shaded regions denote a 95% CI.

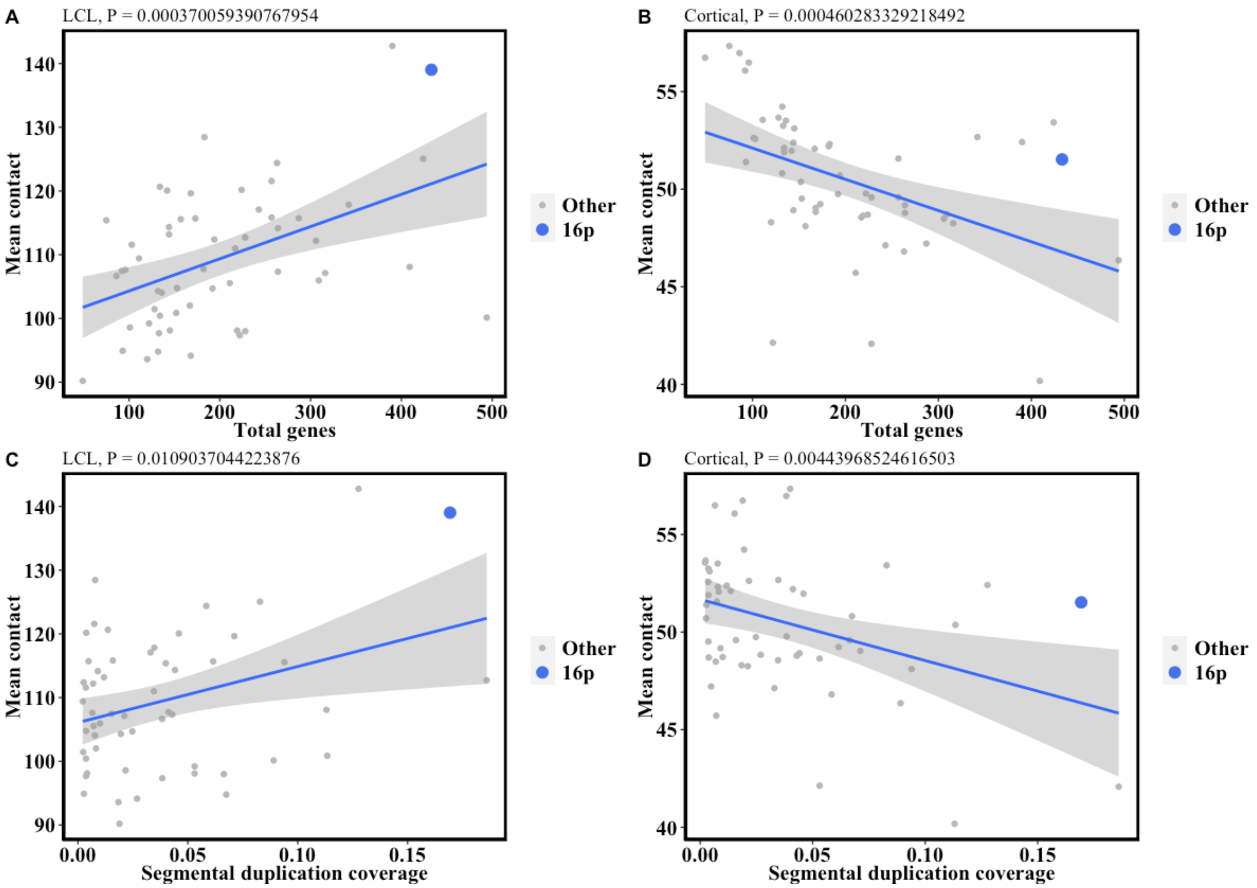

Supplementary Figure 23: Association between segmental duplication content / gene density and chromatin contact

The relationship between gene density, segmental duplication content and mean contact frequency in two Hi-C datasets, one of lymphoblastoid cell line and one of mid-gestational cortical plate (Methods). Each point is a 33Mb partition (see Methods). Mean contact is the mean within-partition off-diagonal value from the contact matrix. Segmental duplication coverage is defined in Methods. The above-plot P-value is from the Pearson correlation between the x and y variables (two-sided). Shaded gray regions denote 95% CI.

Supplementary Note 1: Derivation of pTDT for case-pseudocontrol genotypes

The genotypes for PGC trios are imputed as cases and pseudocontrols, where pseudocontrol genotypes are constructed from the untransmitted alleles. Below we derive a pTDT estimator using cases and pseudocontrols, instead of the traditional input of cases and both parental genotypes.

pTDT is defined as the standardized difference between case PGS and the expectation defined by the mid-parent PGS:

$$pTDT = \frac{PGS_{case} - PGS_{MP}}{SD(PGS_{MP})}$$

The union of the genotypes of the case and the pseudocontrol constitute the transmitted and untransmitted alleles from the parents. Thus, the union of the case and pseudocontrol genotypes equals the union of the parental genotypes. Thus, the union of the case and pseudocontrol genotypes divided by 2 equals the average parental genotype, and by extension, the mid-parent PGS:

$$PGS_{MP} = \frac{PGS_{case} + PGS_{pseudocontrol}}{2}$$

Thus, by substitution of the expression for PGS_{MP} into the pTDT equation above, we derive an estimator compatible with case-pseudocontrol genotypes.

Pathway class	Enrichment pathway	Fold enrichment	Enrichment P-value (uncorrected, Fisher's exact test)	Genes
Biological process	Anatomical structure morphogenesis	0.4	5.1e-6	ITGAX, TAOK2, AXIN1, ARMC5, MYH11, NPRL3, TNFRSF12A, BMERB1, MAPK3, PLA2G10, SOX8, PKD1, CREBBP, TBX6, IFT140, TGFB1I1, ROGDI, PALB2, CACNA1H, NTN3, MYLPF, TSC2.
Molecular function	Fatty-acyl-CoA synthase activity	29.4	5.0e06	ACSM1, ACSM3, ACSM2A, ACSM5, ACSM2B
Molecular function	Butyrate-CoA ligase activity	22.9	1.2e-5	ACSM1, ACSM3, ACSM2A, ACSM5, ACSM2B
Molecular function	Medium-chain fatty acid-CoA ligase activity	22.9	1.2e-5	ACSM1, ACSM3, ACSM2A, ACSM5, ACSM2B
Cellular component	Hemoglobin complex	18.7	2.6e-5	HBZ, HBM, HBA2, AHSP, HBQ1

Supplementary Table 1: Bonferroni significant enrichments using gene ontology analysis of genes on 16p.

We performed gene ontology (GO) analysis to evaluate enrichment of genes on 16p in annotated biological pathways (http://geneontology.org/). We used the same 17,909 genes from the gene density analysis as reference genes. We tested for enrichment of all genes on 16p (midpoint < 33,000,000 bp, n = 433 genes) across three classes of annotations: biological process, molecular function, and cellular component. The GO analysis for molecular function and cellular component returned multiple bonferroni-significant enrichments: multiple lipid/fatty acid pathways (Fatty-acyl-CoA synthase activity, Butyrate-CoA ligase activity, Medium-chain fatty acid-CoA ligase activity, >20x enrichment for each), and hemoglobin complex (19x enrichment). The lipid/fatty acid pathways return an enrichment because there are 5 acyl-CoA-synthase genes located within 500kb of each other on 16p around Mb 20. Similarly, the hemoglobin complex pathway returns an enrichment because 4 hemoglobin subunits are clustered together within 100kb of each other at the start of chromosome 16. These examples raise a critical point: since functionally similar genes are often clustered together on the genome[5], a gene set enrichment signal will be dominated by whichever functional cluster of genes happens to be located within the region of interest. Thus, we do not believe that canonical gene set enrichment approaches are suited to regional enrichment analysis. It is also possible that decreased expression across 16p does not exert direct phenotypic effect, but instead propagates to interact with gene/protein networks elsewhere in the cell or cellular network. As cell-type specific interaction networks become available, we look forward to integrating with our analyses. The P-value is from a Fisher's exact test.

Name	Cases	Controls	Total samples	SNPs after LDpred	Reference
iPSYCH ASD	19870	39078	58948	1152500	iPSYCH consortium
iPSYCH+PGC ASD	26067	46455	72522	1169771	iPSYCH, PGC consortium
iPSYCH ADHD	25895	37148	63043	1090116	iPSYCH consortium

Supplementary Table 2: GWAS summary statistics.
We used multiple GWAS to construct polygenic risk scores. We used the iPSYCH-only ASD PGS in multiple analyses to avoid overlap with target samples (SSC, PGC) included in the iPSYCH+PGC GWAS. SNPs after LDpred refers to the number of markers in the whole-genome polygenic risk score after processing with LDpred.

**ASD family cohorts (European ancestry)**	***Complete families***	***Unique families***	**Access**
SSC (parents and proband)	1851	1851	SFARI
SSC (parents and unaffected sibling)	1509	1509	SFARI
SPARK (multiplex families, parent cases removed)	3197	2880 (2596 with 1 proband, 253 with 2, 29 with 3, 2 with 4)	SFARI
Psychiatric Genomics Consortium (multiplex families)	4335	3258 (2312 with 1 proband, 829 with 2 probands, 104 with 3, 12 with 4, 1 with 5)	PGC
**ADHD family cohorts (European ancestry)**	***Complete families***	***Unique families***	**Access**
Psychiatric Genomics Consortium (parents and proband)	1634	1634	PGC
**Genotyped-expression cohorts**	**Total n**	**Schizophrenia diagnosis (n)**	**Access**
Dropulation donors, European ancestry	122	58	McCarroll Lab
CommonMind, African ancestry	193	67	CommonMind/Synapse
CommonMind, European ancestry	229	68	CommonMind/Synapse

Supplementary Table 3: Cohorts used in analysis.
We used multiple cohorts for genotype-phenotype and genotype-expression analyses. Abbreviations: SSC (Simons Simplex Collection), SPARK (Simons Foundation Powering Autism Research), Dropulation (McCarroll Lab single-nucleus RNA-sequencing dataset, see Ling et al. (forthcoming) for details).

Partition number	Chromosome	Start position (BP)	End position (BP)
1	1	11008	33011008
2	1	33011008	66011008
3	1	66011008	99011008
4	1	99011008	132011008
5	1	132011008	165011008
6	1	165011008	198011008
7	1	198011008	231011008
8	2	10188	33010188
9	2	33010188	66010188
10	2	66010188	99010188
11	2	99010188	132010188
12	2	132010188	165010188
13	2	165010188	198010188
14	2	198010188	231010188
15	3	60197	33060197
16	3	33060197	66060197
17	3	66060197	99060197
18	3	99060197	132060197
19	3	132060197	165060197
20	4	10253	33010253
21	4	33010253	66010253
22	4	66010253	99010253
23	4	99010253	132010253
24	4	132010253	165010253
25	5	10056	33010056
26	5	33010056	66010056
27	5	66010056	99010056
28	5	99010056	132010056
29	5	132010056	165010056

30	6	63979	33063979
31	6	33063979	66063979
32	6	66063979	99063979
33	6	99063979	132063979
34	6	132063979	165063979
35	7	16864	33016864
36	7	33016864	66016864
37	7	66016864	99016864
38	7	99016864	132016864
39	8	11774	33011774
40	8	33011774	66011774
41	8	66011774	99011774
42	8	99011774	132011774
43	9	12704	33012704
44	9	33012704	66012704
45	9	66012704	99012704
46	9	99012704	132012704
47	10	60684	33060684
48	10	33060684	66060684
49	10	66060684	99060684
50	10	99060684	132060684
51	11	128196	33128196
52	11	33128196	66128196
53	11	66128196	99128196
54	11	99128196	132128196
55	12	60317	33060317
56	12	33060317	66060317
57	12	66060317	99060317
58	12	99060317	132060317
59	13	19020095	52020095
60	13	52020095	85020095

61	14	19000059	52000059
62	14	52000059	85000059
63	15	20000447	53000447
64	15	53000447	86000447
65	16	60288	33060288
66	16	33060288	66060288
67	17	828	33000828
68	17	33000828	66000828
69	18	10854	33010854
70	18	33010854	66010854
71	19	69984	33069984
72	20	61098	33061098
73	21	9412099	42412099
74	22	16050840	49050840

Supplementary Table 4: 33Mb partitions used in primary analysis.
See Methods for details on how partitions were defined.

References

1. Satterstrom, F. K. *et al.* Large-Scale Exome Sequencing Study Implicates Both Developmental and Functional Changes in the Neurobiology of Autism. *Cell* **180**, 568–584.e23 (2020).
2. Finucane, H. K. *et al.* Heritability enrichment of specifically expressed genes identifies disease-relevant tissues and cell types. *Nat. Genet.* **50**, 621–629 (2018).
3. Chang, C. C. *et al.* Second-generation PLINK: rising to the challenge of larger and richer datasets. *GigaScience* vol. 4 (2015).
4. Price, A. L. *et al.* Long-range LD can confound genome scans in admixed populations. *American journal of human genetics* vol. 83 132–5; author reply 135–9 (2008).
5. Andrews, T. *et al.* The clustering of functionally related genes contributes to CNV-mediated disease. *Genome Res.* **25**, 802–813 (2015).
