# Supplementary material for: Statistical and functional convergence of common and rare genetic influences on autism at chromosome 16p
Source: Nat Genet. 2022 Oct 24;54(11):1630–9. doi: 10.1038/s41588-022-01203-y (PMC9649437; doi:10.1038/s41588-022-01203-y)
Supplement: Supplementary file 2 — Reporting Summary [file 41588_2022_1203_MOESM2_ESM.pdf]

nature portfolio

Corresponding author(s): Daniel Weiner

Last updated by author(s): Aug 20, 2022

Reporting Summary

Nature Portfolio wishes to improve the reproducibility of the work that we publish. This form provides structure for consistency and transparency in reporting. For further information on Nature Portfolio policies, see our Editorial Policies and the Editorial Policy Checklist.

Statistics

For all statistical analyses, confirm that the following items are present in the figure legend, table legend, main text, or Methods section.

n/a	Confirmed	
☐	☒	The exact sample size (*n*) for each experimental group/condition, given as a discrete number and unit of measurement
☒	☐	A statement on whether measurements were taken from distinct samples or whether the same sample was measured repeatedly
☐	☒	The statistical test(s) used AND whether they are one- or two-sided<br>*Only common tests should be described solely by name; describe more complex techniques in the Methods section.*
☐	☒	A description of all covariates tested
☐	☒	A description of any assumptions or corrections, such as tests of normality and adjustment for multiple comparisons
☐	☒	A full description of the statistical parameters including central tendency (e.g. means) or other basic estimates (e.g. regression coefficient) AND variation (e.g. standard deviation) or associated estimates of uncertainty (e.g. confidence intervals)
☐	☒	For null hypothesis testing, the test statistic (e.g. *F*, *t*, *r*) with confidence intervals, effect sizes, degrees of freedom and *P* value noted<br>*Give P values as exact values whenever suitable.*
☒	☐	For Bayesian analysis, information on the choice of priors and Markov chain Monte Carlo settings
☒	☐	For hierarchical and complex designs, identification of the appropriate level for tests and full reporting of outcomes
☐	☒	Estimates of effect sizes (e.g. Cohen's *d*, Pearson's *r*), indicating how they were calculated

Our web collection on statistics for biologists contains articles on many of the points above.

Software and code

Policy information about availability of computer code

Data collection	No data was collected for primary use in this manuscript
Data analysis	Data was analyzed using R v4.1 and Python v3.7

For manuscripts utilizing custom algorithms or software that are central to the research but not yet described in published literature, software must be made available to editors and reviewers. We strongly encourage code deposition in a community repository (e.g. GitHub). See the Nature Portfolio guidelines for submitting code & software for further information.

Data

Policy information about availability of data

All manuscripts must include a data availability statement. This statement should provide the following information, where applicable:

- Accession codes, unique identifiers, or web links for publicly available datasets
- A description of any restrictions on data availability
- For clinical datasets or third party data, please ensure that the statement adheres to our policy

Individual-level genotypes are available via request to the Simons Foundation Autism Research Initiative (sfari.org) and from the Psychiatric Genomics Consortium (https://pgc.unc.edu/) and its contributing data holders. GWAS summary statistics from iPSYCH are available by request from the members of the consortium. RNA-sequencing data is available from repository 2304 at the National Database for Autism Research and the CommonMind Consortium (https://www.nimhgenetics.org/resources/commonmind). Hi-C data is available via the respective referenced publications. GTEx specific expression data is available from the Price Lab repository

(https://alkesgroup.broadinstitute.org/LDSCORE/LDSC_SEG_ldscores/tstats/). Additional gene information is available from the gnomAD browser (https://gnomad.broadinstitute.org/downloads).

Human research participants

Policy information about studies involving human research participants and Sex and Gender in Research.

Reporting on sex and gender	Sex and gender-based analyses were not performed.
Population characteristics	Characteristics are available in Supplementary Table 3
Recruitment	No participants were specifically recruited for this study.
Ethics oversight	We confirm that this study was reviewed and approved by Partners Human Research of Partners HealthCare. The study name is Molecular Study of Cognitive and Behavioral Variation (IRB: 2015P002376). The Principal Investigator is Elise Robinson. The iPSYCH study was approved by the Danish Data Protection Agency and the Scientific Ethics Committee in Denmark.

Note that full information on the approval of the study protocol must also be provided in the manuscript.

Field-specific reporting

Please select the one below that is the best fit for your research. If you are not sure, read the appropriate sections before making your selection.

☒ Life sciences ☐ Behavioural & social sciences ☐ Ecological, evolutionary & environmental sciences

For a reference copy of the document with all sections, see nature.com/documents/nr-reporting-summary-flat.pdf

Life sciences study design

All studies must disclose on these points even when the disclosure is negative.

Sample size	The largest sample sizes from the available data were used throughout the analysis.
Data exclusions	No data were excluded from analysis.
Replication	All experiments were replicated when independent data was available, including: S-pTDT identification of 16p (SSC+SPARK; PGC), regional PGS association to expression (single-nucleus RNA-seq data; bulk RNA-seq data); chromatin contact analysis (LCL; cortical plate). No replication attempts failed.
Randomization	Randomization was not relevant to this study since no interventions were performed.
Blinding	Blinding was not relevant to this study since no interventions were performed.

Reporting for specific materials, systems and methods

We require information from authors about some types of materials, experimental systems and methods used in many studies. Here, indicate whether each material, system or method listed is relevant to your study. If you are not sure if a list item applies to your research, read the appropriate section before selecting a response.

Materials & experimental systems

n/a	Involved in the study	
☒	☐	Antibodies
☒	☐	Eukaryotic cell lines
☒	☐	Palaeontology and archaeology
☒	☐	Animals and other organisms
☒	☐	Clinical data
☒	☐	Dual use research of concern

Methods

n/a	Involved in the study	
☒	☐	ChIP-seq
☒	☐	Flow cytometry
☒	☐	MRI-based neuroimaging
